# By recruiting HDAC1, MORC2 suppresses p21Waf1/Cip1 in gastric cancer

**DOI:** 10.18632/oncotarget.3889

**Published:** 2015-05-08

**Authors:** Qing Zhang, Yanyan Song, Wei Chen, Xiaohui Wang, Zhifeng Miao, Liu Cao, Feng Li, Guiling Wang

**Affiliations:** ^1^ Department of Cell Biology, Key Laboratory of Cell Biology, Ministry of Public Health, and Key Laboratory of Medical Cell Biology, Ministry of Education, China Medical University, Shenyang, China; ^2^ Department of Surgical Oncology and General Surgery, First Hospital of China Medical University, Shenyang, China

**Keywords:** MORC2, p21, HDAC1, cell proliferation, gastric cancer

## Abstract

Microrchidia (MORC) family CW-type zinc-finger 2 (MORC2) regulates chromatin remodeling during the DNA-damage response, represses gene transcription, promotes lipogenesis. Here, we found that MORC2 down-regulated p21 by recruiting HDAC1 to the p21 promoter, in a p53-independent manner. MORC2-mediated down-regulation of p21 in turn promoted cell cycle progression in gastric cancer cells. Furthermore, MORC2 expression correlated negatively with p21 expression in gastric tumors in patients. We suggest that MORC2 may be a potential therapeutic target in cancer.

## INTRODUCTION

Gastric cancer is the second-third most common cause of cancer-related death in the world [1]. To improve cancer patient survival, it is a central event to investigate the proteins governing development and progression of gastric cancer. Among the proteins controlling cell cycle progression, the cyclin-dependent kinase (CDK) inhibitor p21^Waf/Cip1^ (referred to as p21 hereafter) has been considered as key regulator of cell proliferation and survival [2] through inhibiting the activity of several cyclin-CDK complexes to induce cell cycle arrest [3, 4].

Human MORC2 (microrchidia family CW-type zinc-finger 2), also known as KIAA0852, ZCW3 or ZCWCC1, containing a CW-type zinc-finger and three coiled-coil domains [5], is a member of the MORC protein family and mainly localizes in the nucleus [6]. Recent studies revealed that MORC2 promoted chromatin remodeling during the DNA-damage response [7] and regulated lipogenesis [8]. However, its function remains largely unknown.

In our previous study, we found that MORC2 may act as a transcriptional repressor and play a role in cancer [6, 9], which promoted us to identify the target gene of MORC2 underlying the mechanism in cancer. Thus, we carried out a microarray experiment and found a lot of MORC2 target genes, among which p21 is down-regulated markedly in stable overexpressing of MORC2 SGC-7901 cells compared to vector control. A series of assays were performed and showed that over-expression of MORC2 down-regulated p21 expression, which is involved in HDAC1 modification. Furthermore, our results indicated that MORC2-mediated p21 down-regulation promotes gastric cell proliferation. Furthermore, MORC2 negatively correlates with p21 expression in gastric cancer samples, suggesting that MORC2 might be as a potential therapeutic target for cancer.

## RESULTS

### MORC2 downregulates p21 expression

**Figure 1:**
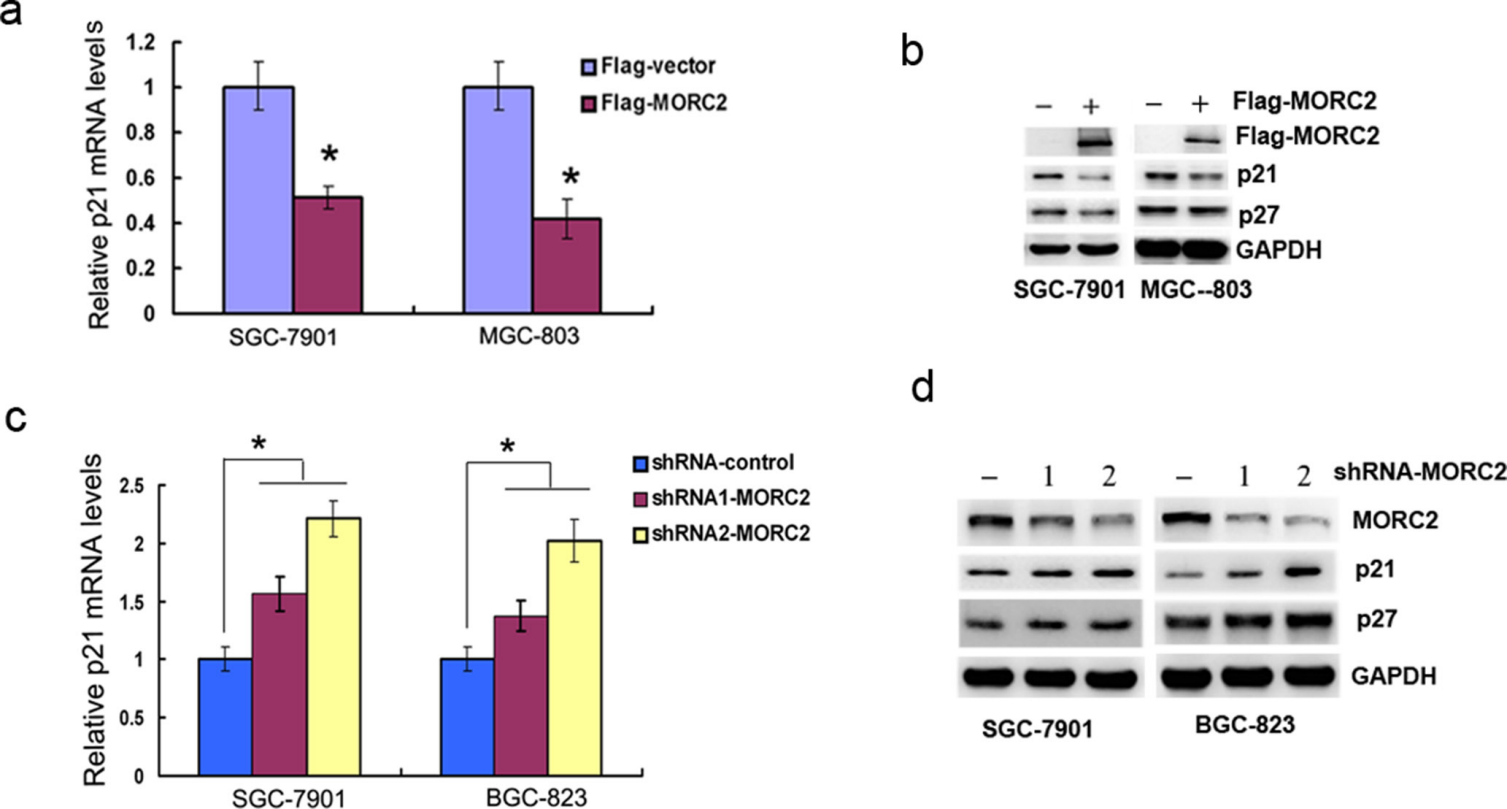
MORC2 down-regulates p21 expression **a–b.** Overexpression of MORC2 down-regulates p21 mRNA and protein expressions. Flag-MORC2 or vector control was transiently transfected into SGC-7901 cells (*left panel*) and MGC-803 cells (*right panel*). (a) The mRNA levels were estimated by qPCR analysis. Values are means ± SD (*n* = 3), *P < 0.05. (b) The protein levels were analyzed by western blot analysis. GAPDH was used as a control. **c–d.** Specific knockdown of MORC2 up-regulates p21 mRNA and protein expressions. Endogenous MORC2 in SGC-7901 cells (*left panel*) and BGC-823 cells (*right panel*) was knocked down by two different shRNAs (#1 and #2) targeting MORC2 by lentivirus infection. (c) The p21 mRNA levels were estimated by qPCR analysis. Values are means ± SD (*n* = 3), *P < 0.05. (d) The p21 protein levels were analyzed by western blot analysis. The efficacy of MORC2 shRNAs was demonstrated by depletion of MORC2. GAPDH was used as a control.

To uncover new function of MORC2, we carried out a microarray experiment and found a lot of MORC2 target genes which are involved in a variety of biological functions including cell cycle and cell apoptosis, material transport and metabolism, cell adhesion and cell motility, immune response and etc. Most of these MORC2 target genes were down-regulated, among which p21 is down-regulated markedly in Flag-MORC2/SGC-7901 cells (an exogenous MORC2 stable expression gastric cancer cell line) compared to vector control. Then we performed qPCR and western blot assays to confirm the result. Our results showed that the mRNA and protein levels of p21 were reduced with the ectopic MORC2 expression in SGC-7901 cells and MGC-803 cells (Figure 1a and 1b), depletion of the endogenous MORC2 by specific shRNA resulted in an increase of p21 expression in lentivirus infection SGC-7901 cells and BGC-823 cells, but has little effect on the protein level of p27 (Figure 1c and 1d). These results suggest that MORC2 specifically represses p21 expression.

### The repression of p21 by MORC2 is not related with p53 status in gastric cancer cells

**Figure 2:**
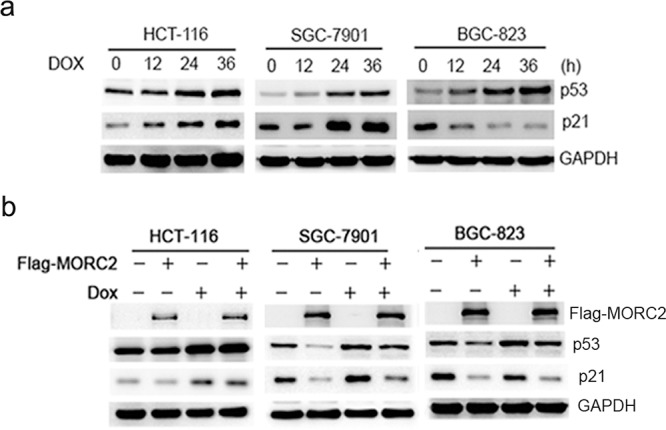
The repression of p21 by MORC2 is not related with p53 status in gastric cancer cells **a.** To treat these gastric cancer cells with doxorubicin (DOX, DNA damage inducer) treatment for 36 hours (400 ng/ml) to induce p53 accumulation in a time-dependent manner, the wild type p53 of HCT-116 colon cancer cells were used as control. The lysates were probed with indicated antibodies. **b.** The ecotopic MORC2 can downregulate p21 expression in both SGC-7901 and BGC-823 cells. These cells transiently transfected into the ecotopic MORC2 with and without DOX treatment for 36 hours (400 ng/ml) to induce p53 accumulation, the wild type p53 of HCT-116 colon cancer cells were used as control. The lysates were probed with indicated antibodies.

P53 is one of the most frequently mutated genes in gastric cancer and one of its target genes is p21. To determine that the repression of p21 is caused by MORC2 rather than mutant p53, we treated cells with doxorubicin (Dox, DNA damage inducer) to induce p53 accumulation in a time-dependent manner. The wild type p53 of HCT-116 colon cancer cells were used as control. Our results indicated that Dox treatment resulted in an increase of p21 expression in both HCT-116 cells and SGC-7901 cells, and a reduction of p21 was shown in BGC-823 cells (Figure 2a), which suggest that SGC-7901 cells are wild type p53, and BGC-823 cells are mutant p53. Moreover, we transfected ectopic MORC2 into the wild type p53 of SGC-7901 cells and mutant p53 of BGC-823 cells with or without Dox treatment, these results indicated that the levels of p21 are down-regulated (Figure 2b). Therefore, our results suggest that the repression of p21 is due to MORC2 rather than mutant p53 in gastric cancer cells.

### MORC2 can bind to p21 promoter and repress its activity

**Figure 3:**
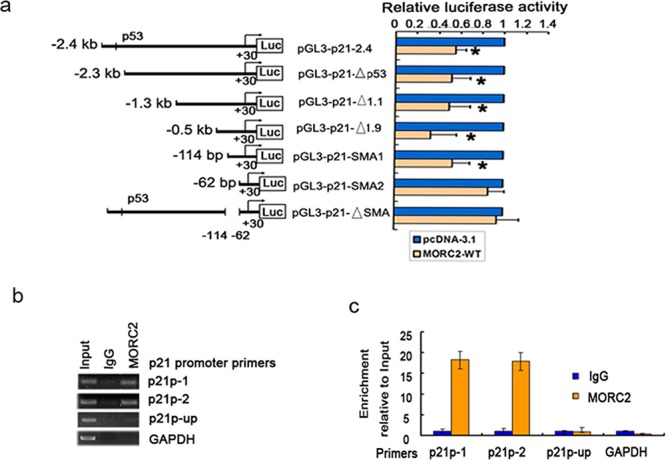
MORC2 can bind to p21 promoter and repress its activity **a.** The region (−114 bp to −62 bp) in p21 promoter is important for the suppression function of MORC2 on p21 transcription. (*left pane*l) Schematic representation of the 2.4 kb full-length or several truncations and mutant of human p21 promoter fusions to luciferase were constructed and are diagramed. (*right panel*) SGC-7901 cells were serially transfected with various p21 promoters deletion constructs indicated in Figure 2a (left panel) and with or without His-MORC2 expression plasmid as indicated. Luciferase activities were determined and normalized to Renilla activity 24 h after transfection. Results are expressed as a percentage of the MORC2-untransfected control that is taken as 100%. *P < 0.05 compared with control. **b.** ChIP DNA analysis of MORC2 binding to p21 promoter. Primer sets probing the proximal region of the p21 promoter were used p21-1 and p21-2, as were primers probing a region of the p21 promoter 4 Kb upstream from the transcriptional start site (p21-up) or the GAPDH promoter. DNA content after immunoprecipitation with MORC2 antibody or nonspecific antibody (IgG) controls by PCR amplification and 1.5% agarose gel electrophoresis. **c.** ChIP analysis of MORC2 binding to the endogenous p21 promoter. DNA content after immunoprecipitation with MORC2 antibody or nonspecific antibody (IgG) controls, were determined by qPCR with indicated primers. All values were expressed relative to Input DNA content.

Next step was to determine which regions are required for the repression function of MORC2 on p21 transcription. A series of 5′ promoter deletion mutants of the p21 promoter [10] proximal to the transcriptional initiation site were transfected into SGC-7901 cells (Figure 3a, *left panel*). The results showed that these p21 promoters activity (containing p21P, p21PΔ53, p21PΔ1.1, p21PΔ1.9, p21SMA1) were repressed by overexpressed MORC2 compared with vector control, whereas the p21SMA2 promoter (containing only 62 bp) activity was abolished by MORC2. Furthermore, MORC2 repressing p21 promoter activity was eliminated when this segment of p21ΔSMA between the −114 bp (p21SMA1 construct) and −62 bp (p21SMA2 construct) was removed from the p21 full-length promoter (Figure 3a, *right panel*). These results indicate that overexpressed MORC2 repressing p21 promoter activity requires the sequence between −114 bp and −62 bp in p21 promoter. We then performed ChIP experiments with MORC2 antibody to demonstrate endogenous MORC2 binding to p21 promoter. We designed two independent primer sets (p21p-1 and p21p-2), including the sequence between −114 bp and −62 bp in p21 promoter, which contains HDAC1 binding sites and was efficient in amplifying ChIP DNA in quantitative real-time PCR (Q-ChIP). As a control, we also designed primers at 4 Kb upstream from the transcriptional start site (p21p-up) of the p21 promoter and of the GAPDH promoter. As shown in Figure 3b, enrichment of MORC2 binding to p21p-1 and p21p-2 promoter, respectively, was observed in comparison with IgG by PCR amplification of ChIP DNA, whereas there was no such enrichment of MORC2 binding to p21p-up promoter, or to the GAPDH promoter. Similar results were obtained by Q-ChIP (Figure 3c). Therefore, these results suggest that endogenous MORC2 can bind to the region (−114 bp to −62 bp) in p21 promoter to repress its activity.

### MORC2 recruits HDAC1 to bind p21 promoter and repress its activity

**Figure 4:**
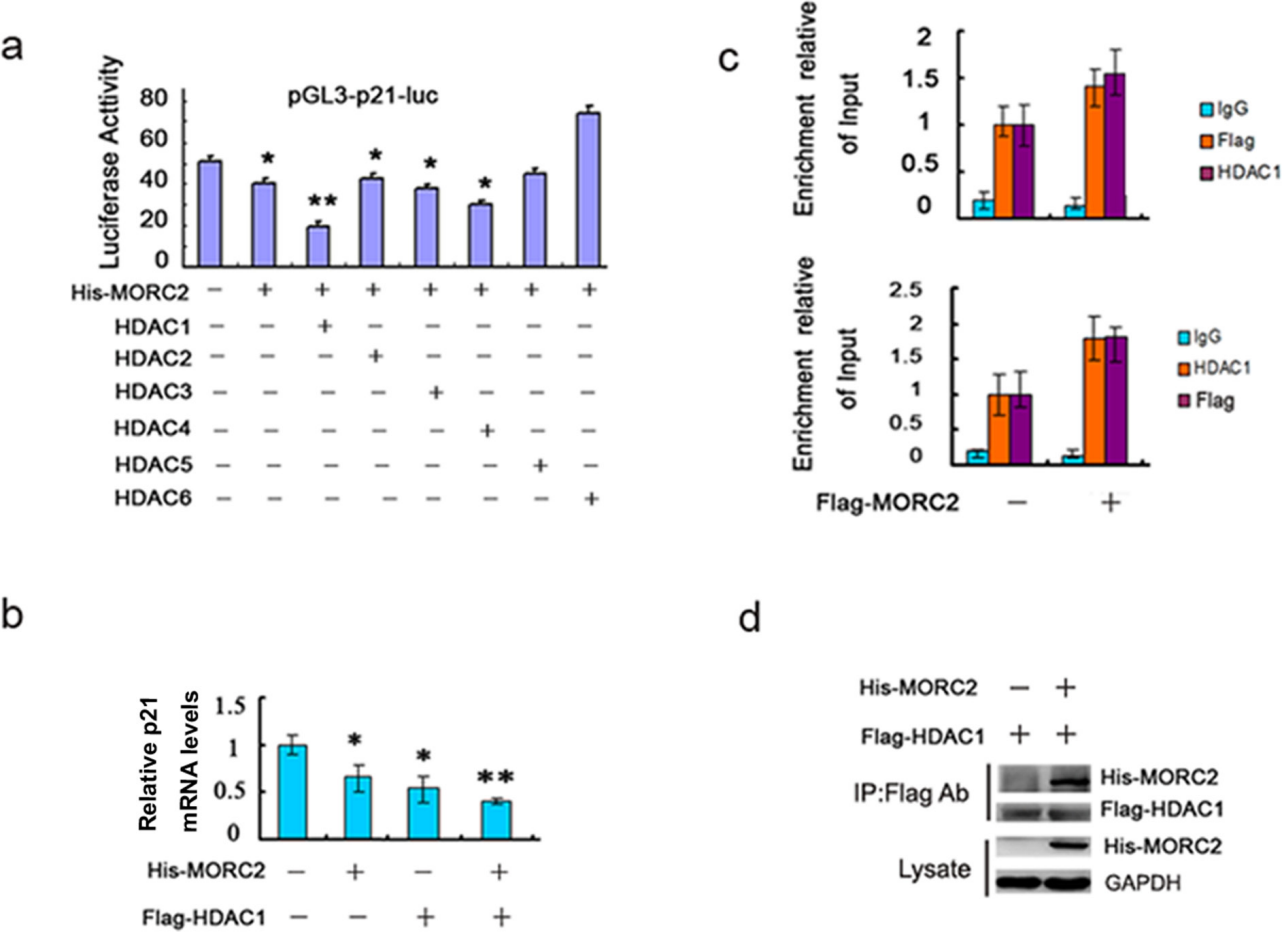
MORC2 recruits HDAC1 to repress p21 promoter activity **a.** The histone deacetylases expression vector and Flag-MORC2 along with pGL3-p21-luc reporter plasmid were transiently co-transfeced into SGC-7901 cells and analyzed for luciferase activity. Luciferase activities were determined and normalized to pRL-TK (Renilla) activity 24 h after transfection. Results are shown as fold induction relative to that of the cells transfected without HDAC plasmid and are the means ± SD from at least three individual experiments. *P < 0.05, **P < 0.01. **b.** Flag-MORC2 and HDAC1 were transiently transfected into SGC-7901 cells as indicated, and the mRNA level was estimated by qPCR analysis. Values are means ± SD (*n* = 3), *P < 0.05, **P < 0.01. **c.** Sequential ChIP assays analyse MORC2 HDAC1 recruitment to p21 promoter. The SGC-7901 cells were transfected with Flag-MORC2, conducting initial immunoprecipitation with anti-Flag antibody, followed by a second immunoprecipitation with IgG or anti-HDAC1 (*upper panel*), or initial immunoprecipitation with anti-HDAC1 followed by a second immunoprecipitation with IgG or anti-Flag antibody (*down panel*). All values were initially expressed relative to relevant Input DNA content. **d.** SGC-7901 cells were co-transfected with Flag-HDAC1 and His-MORC2 or control vector as indicated, and performed to immunoprecipitate with anti-Flag antibody, and precipitates were immunoblotted with His-tagged antibody. The lysate was probed with the indicated antibodies (*bottom panels)*.

Previous studies have demonstrated that the class I and II histone deacetylases (HDACs) [11], including HDAC1 [12, 13], HDAC2 [14], HDAC3 [15] and HDAC4 [16], repress p21 expression in multiple human cancers. We further tested the effect of the HDACs together with Flag-MORC2 on p21 transcription activity. The results indicated that the HDAC1 together with MORC2 exerted distinct repressive effects on the p21 promoter activity (Figure 4a). To further investigate how the mRNA level of p21 was affected by MORC2 and HDAC1, we performed qPCR experiments and showed that HDAC1 together with MORC2 had much more strongly repressive role in p21 mRNA level than individual (Figure 4b).

To demonstrate whether the binding of MORC2 to p21 promoter recruits HDAC1, we performed sequential ChIP experiments with the p21p-2 primer. Flag-MORC2 significantly recruits HDAC1 to p21 promoter (Figure 4c, *upper panel*), when anti-HDAC1 immunoprecipitation was performed on eluted chromatin obtained from an initial anti-Flag immunoprecipitation. Likewise, similar results were also observed by the reverse sequence of ChIP experiments (Figure 4c, *down panel*). To confirm the MORC2-HDAC1 association *in vivo*, we carried out immunoprecipitation and found that MORC2 strongly bound to HDAC1 compared to control vector (Figure 4d). Therefore, those results suggest that the MORC2-mediated down-regulation of p21 involves HDAC1 recruitment.

### MORC2-mediated p21 repression promotes gastric cancer cell proliferation

**Figure 5:**
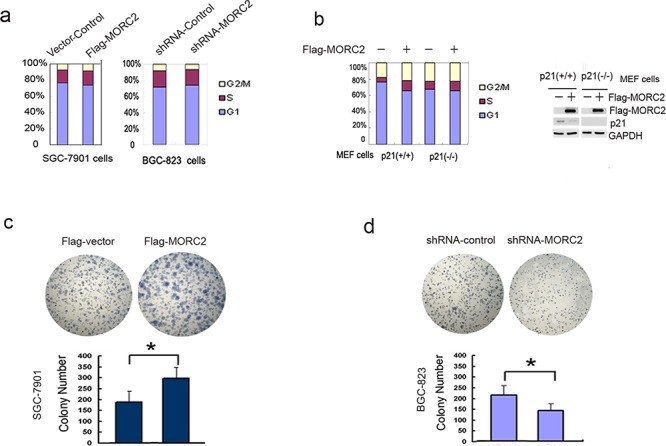
MORC2-mediated p21 repression promotes cell proliferation (**a.** *left panel*) Overexpression of MORC2 promotes cell cycle progression. The lentivirus-mediated Flag-MORC2/SGC-7901 cells were cultured and stained with propidum iodide. Cell cycle distribution was measured by FACS. Values are the means ± SD from three individual experiments. (**a.** *right panel*) shRNA-MORC2 inhibits cell cycle progression. The lentivirus-mediated shRNA-MORC2 BGC-823 cells were cultured and stained with propidum iodide. Cell cycle distribution was measured by FACS. Values are the means ± SD from three individual experiments. **b.** FCM experiments analysis of cell cycle alterations induced by MORC2 in p21-deficient cells. The lentivirus-mediated Flag-MORC2 of p21 MEF cells were cultured, and stained with propidum iodide. Cell cycle distribution was measured by FACS. Values are the means ± SD from three individual experiments. The lentivirus-mediated Flag-MORC2 of p21 MEF cells were cultured and subjected to Western blotting analysis. Cell extracts were probed with indicated antibodies. **c–d.** The expression level of MORC2 affects colony formation. Colony formation assays were performed with the stable expressing lentivirus-mediated Flag-MORC2/SGC-7901 cells and shRNA-MORC2/BGC-823 cells. Representative results are shown.

To examine whether p21 repression by MORC2 affects cell cycle progression, we performed flow cytometry assays and found that Flag-MORC2/SGC-7901 cells showed significant decrease in the percentage of G1 phase cells, and significant increase in the percentage of S and G2/M phase compared with vector control cells (Figure 5a, *left panel*). Then, to analysis the effect of MORC2 siRNA on cell cycle progression, shRNA-MORC2/BGC-823 cells increased the percentage of G1 phase cells, and decreased the percentage of S phase cells (Figure 5a, *right panel*), Taken together, these data suggest that MORC2-mediated p21 repression can promote cell cycle progression.

To confirm that cell cycle alterations induced by MORC2 are mediated by p21, we repeated the FCM experiments with p21-deficient MEF cells [17]. In p21-(+/+) MEF cells, Flag-MORC2 showed significant decrease in the percentage of G1 phase, and significant increase in the percentage of S phase compared with vector control (Figure 5b, *left panel*); while in p21-deficient (−/−) MEF cells, Flag-MORC2 did not show cell cycle alterations compared with vector control (Figure 5b, *right panel*). Our findings therefore, suggest that MORC2-induced cell cycle alteration is mediated by p21.

In order to confirm the role of MORC2 in gastric cancer cells, colony formation assays were performed with Flag-MORC2/SGC-7901 cells and shRNA-MORC2/BGC-823 cells. As shown in Figure 5c and Figure 5d, stable overexpressing of MORC2 cells formed more and larger colonies *in vitro* compared with vector control, while BGC-823 cells with stable expressing shRNA-MORC2 formed less and smaller colonies compared with shRNA-control. Taken together, overexpression of MORC2 contributes to cell cycle progression and proliferation of gastric cancer cells.

### MORC2 negatively correlates with p21 expression in gastric cancer samples

**Figure 6:**
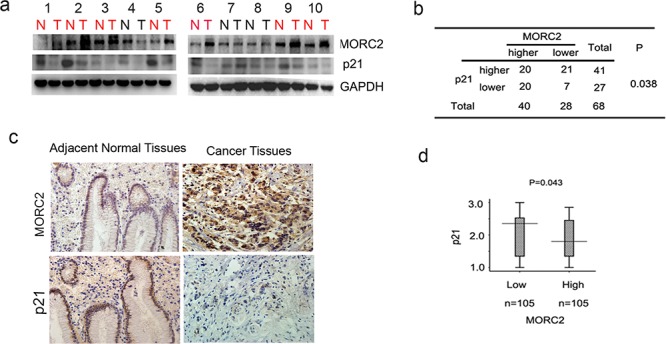
MORC2 negatively correlates with p21 expression in gastric cancer samples **a.** Evaluate the expression of the indicated proteins in clinical tissues by western Blot. Lysates of 68 tumor tissues (T) and matched adjacent normal tissues (N) pairs were analyzed by Western Blot. Number of cancers with reduced or increased levels of indicated protein relative to normal adjacent tissues and analyze with GAPDH as the reference. The representative 10 pairs were shown. **b.** Summary of the expression in tissues in (a) is shown, with tissues categorized by lower and higher expression. The expression of MORC2 and p21 was analyzed with GAPDH as the reference. In each N and T pair, the lower/higher expression in T, compared with N, is categorized as lower/higher expression. The *P* value was generated using the chi-square test. **c.** Representative images of immunohistochemical staining of MORC2 and p21 expression from one case were shown. Original magnification, ×200. **d.** Statistical analysis of MORC2 and p21 expressions in 210 gastric cancer tissues. Intensity values were expressed as HSCOREs. Box plot of MORC2 and p21 expressions were shown. The subjects were divided into two groups based on MORC2 expression scores in the 210 gastric cancer samples, representing low and high expression. The MORC2 and p21 expression scores were shown as box plots, with the horizontal lines representing the median; the bottom and top of the boxes representing the 25th and 75th percentiles, respectively; and the vertical bars representing the range of data. And extreme cases were marked with a dot. Data was analyzed by one-way analysis of variance (ANOVA) test with Games-Howell's correction.

To show the correlation between MORC2 and p21 in gastric cancer samples, we tested the expressions MORC2 and p21 in freshly frozen gastric cancer tissues and matched adjacent normal tissues from 68 gastric tumor patients. Western blot showed that MORC2 was high expression in 58% (40 of 68) of gastric tumor samples, of which p21 was down-regulated in 50% (20 of 40). Representative samples were shown in Figure 6a. Statistical analysis revealed MORC2 expression negatively correlated with p21 expression (*P* = 0.038; Figure 6b) in gastric cancer tissues.

After that, Immunohistochemical staining for MORC2 and p21 was also performed using sequential sections from the same tissue. To better understand the correlation between them, we divided tumor samples into two groups on the basis of MORC2 amounts (cut off at the median score). The expression levels of MORC2 by immunohistochemistry are correlated with that by western blotting (Figure 6c). The data showed the expression scores of MORC2 showed a negative correlation with p21 amounts (*P* = 0.043; Figure 6d), which indicated that MORC2-mediated p21 expression may contribute to a therapeutic strategy for tumorigenesis.

## DISCUSSION

The transcriptional regulation of p21 has been extensively studied through both p53-dependent [18] and p53-independent mechanisms [19]. The substantial evidence indicated that p21 is clearly up-regulated by other factors acting independently of p53, such as SP1, SP3 [20] and CCAAT/enhancer binding protein-α (C/EBPα) [21]. However, recent studies suggest that, under certain conditions, p21 is often misregulated in human cancers, and its expression is depended on the cellular context and circumstances, indicating that it can act as a tumor suppressor or as an oncogene [3]. Whereas the deregulated expression of p21 in cancer often correlates with the loss of function of transcriptional activators of p21, which may also promotes cancer development. For example, the transcriptional repression of p21 plays a part in the development of tumors in which myc is overexpressed [22].

Expression of p21 has been shown to be up-regulated by the p53 tumor suppressor or gene *in vitro* in response to DNA-damaging agents [19, 23]. However, the repression of p21 is often related with mutated p53 which is in the absence of p53 function. Our results indicated that the repression of p21 expression is due to overexpression of MORC2 rather than mutant p53 in these gastric cancer cell lines. Consistent with observations at the cellular level, 29% (20 of 68) of the gastric cancer patients with MORC2 overexpression examined were in p21 down-regulation by western blot, and we got similar results in immunochemical staining. Collectively, our data indicate that p21 is a novel MORC2 target gene and its expression repression may be at least partially owing to MORC2 overexpression, which contributes to gastric cancer development.

Most reports indicate that HDAC1 repress p53-independent expression of p21 via Sp1-binding sites in the p21 promoter [24, 25]. Here, we found that MORC2 repressed p21 expression and bound to the region (−114 bp to −62 bp) in the p21 promoter covering Sp1 sites, where HDAC1 was also bound to repress p21 expression [12, 15]. Therefore, MORC2-mediated p21 repression is involved in HDAC1 modification in gastric cancer cell lines.

Here, we found that MORC2-mediated p21 expression plays a role in cell cycle progression, cell proliferation and tumorigenicity of gastric cancer cells. Furthermore, MORC2 negatively correlates with p21 expression in clinical gastric cancer. Besides, among the MORC protein family, MORC4 is regarded as a potential lymphoma biomarker [26]. Thus, we speculate that MORC2 may participate in gastric cancer progression and act as a potential therapeutic target for cancer.

## MATERIALS AND METHODS

### Plasmid construction and mutagenesis

The full length and various deletions of p21 promoter-luciferase reporter constructs were generously provided by Dr. Wang XF [10]. Human Flag-HDACs (1-6) expression plasmids were generously provided by Dr E. Seto. The pCDNA3.1-MORC2 (His-MORC2) plasmid was used previously in our paper [6]. Flag-tagged MORC2 were constructed by PCR amplification and sub-cloned into p3 × Flag CMV (Sigma) vector using His-MORC2 plasmid as a template. The p21-deficient MEF cells were provided by Dr. Deng CX [17].

### SiRNA and lentiviral production

pGC-Flag-vector-Lentivirus and pGC-Flag-MORC2-Lentivirus were purchased from Shanghai Gene Chem Company. Stable-overexpression-MORC2, stable-shRNA-MORC2 and control cell lines were selected with puromycin (2 μg/ml) after infection by lentivirus.

### Quantitative real-time PCR, luciferase reporter assay, Immunoprecipitation and western blot

Real-time PCR, luciferase reporter assay, Immunoprecipitation (IP) and western blot have been described previously in detail [27, 28]. The primer sequences of quantitative real-time PCR are provided in Supplementary Table S1.

### Chromatin immunoprecipitation and sequential ChIP

A chromatin immunoprecipitation (ChIP) assay was performed with the EZ-ChIP kit (Upstate Biotechnology). Chromatin samples were immunoprecipitated with MORC2 (Bethyl) antibody. Anti-rabbit IgG (Santa Cruz) was used as a negative control. Nonimmunoprecipitated chromatin fragments were used as an input control. Precipitated DNA was amplified by PCR using primers provided in Supplementary Table S2. LA Taq (TaKaRa) was used to amplify the GC-rich genomic region.

For Sequential ChIP, cross-linked chromatin was immunoprecipitated with an antibody against Flag-tagged as described in Chromatin Immunoprecipitation, except that chromatin was eluted in 10 mM DTT for 30 min at 37°C. Eluted chromatin was diluted 1:50 in buffer subjected to a second immunoprecipitation with antibody against HDAC1, and then eluted with standard elution buffer. A “reverse” sequential ChIP was carried out first with anti-HDAC1 and then with anti-Flag-tagged. Input DNA was calculated from an aliquot of diluted chromatin obtained from the first elution.

### Cell cycle analysis and colony formation assay

Stable overexpressing MORC2 SGC-7901 cell lines were seeded in 60-mm plates to perform the flow cytometry as described in our previous paper [27, 29]. For colony formation assay, 500 cells were plated in six-well plates to assess the proliferation potential of cells and incubated at 37°C in a 5% CO_2_ incubator. After 2 weeks, the number of colonies was counted. Data represent the mean ± SD from 3 independent experiments performed in triplicate wells.

### Tissue samples

Samples of human gastric cancer tissues and paired-adjacent non-tumor gastric tissues further than 5 cm from the tumors were obtained from 210 gastric cancer patients who were underwent gastric resection surgery in the 1st hospital of China Medical University. These gastric cancer tissues and adjacent normal tissues of them were performed to immunohischemical staining. 68 pairs of fresh samples were snap frozen in liquid nitrogen immediately after resection and stored at −80°C until protein extraction for Western Blot. All samples were obtained with patients' informed consent.

The samples were histologically confirmed by staining with hematoxylin-eosin. The histological grade of cancers was assessed according to criteria set by the World Health Organization.

### Immunohistochemistry

Paraffin-embedded gastric tumor tissues were obtained from the First Hospital of China Medical University. Five-micrometer-thick consecutive sections were cut and mounted on glass slides. The slides were deparaffinized, and rehydrated, prior to antigen retrieval, and blocking endogenous peroxidases. The sections were then washed three times in 0.01 mol/L PBS for 5 minutes each and blocked for 1 h in 5% normal goat serum. The sections were exposed to anti-MORC2 (1:100) and anti-p21 (1:200) 4°C overnight. After brief washes in 0.01 mol/L PBS, sections were exposed for 2 h to 0.01 mol/L PBS containing horseradish peroxidase-conjugated goat anti-rabbit immunoglobulin G (1:200), followed by development with 0.003% H_2_O_2_ and 0.03% 3, 3′-diaminobenzidine in 0.05 mol/L Tris-HCl.

All of the immunostained sections were reviewed by two authors who had no knowledge of the patients' clinical status. Five areas selected at random were scored. All sections were scored in a semiquantitative manner according to a previously described method, which reflects both the intensity and percentage of cells staining at each intensity [30]. Intensity was classified as 0 (no staining), +1 (weak staining), +2 (distinct staining), or +3 (very strong staining). A value designated as the ‘HSCORE’ was obtained for each slide by using the following algorithm: HSCORE = ∑ (I × PC), where I and PC represent the staining intensity and the percentage of cells that stain at each intensity, respectively. And the corresponding HSCOREs were calculated separately. The results were evaluated separately by 2 independent observers. Immunohistochemical results were judged by HSCORE [31, 32] (histological score).

### Statistical analysis

All statistical analyses were carried out using the SPSS 16.0 software and the results were considered to be significant when the *P* value was <0.05. Data are presented as mean ± SD from at least three separate experiments. Statistical analysis was performed with Student's *t*-test, non-parametric test (Mann-Whitney *U* test between 2 groups and Kruskall-Wallis test for 3 or more groups). The statistical significance of correlations was calculated by a chi-square test and Spearman's rank correlation.

## SUPPLEMENTARY TABLES


