# Supplementary material for: By recruiting HDAC1, MORC2 suppresses p21Waf1/Cip1 in gastric cancer
Source: Oncotarget. 2015 May 8;6(18):16461–70. doi: 10.18632/oncotarget.3889 (PMC4599282; doi:10.18632/oncotarget.3889)
Supplement: Supplementary file 1 [file oncotarget-06-16461-s001.pdf]

SUPPLEMENTARY TABLES

Supplementary Table S1. Primer Sequences for Quantitative Reverse-Transcription PCR Analysis

Gene	Direction	Sequence (5′to3′)
Human MORC2	Forward	GCAAGCGGGGCAGATTT
	Reverse	CTTGGTGTCGTGTCTGTGGG
Human p21	Forward	GGCAGACCAGCATGACAGATT
	Reverse	GCGGATTAGGGCTTCCTCT
Human GAPDH	Forward	GAAGGTCGGAGTCAACGGAT
	Reverse	CTGGAAGATGGTGATGGGATT

NOTE. Glyceraldehyde-3-phosphate dehydrogenase (GAPDH) was amplified as an internal control.

Supplementary Table S2. Precipitated DNA was amplified by PCR using primer sequences

Human p21 promoter	Direction	Sequence (5′to3′)
p21p-1	Forward	GGCCCAGAGCCGAGCCAAGCGT
	Reverse	CGGCTCAGCTGCTCGCTGTCCA
p21p-2	Forward	GGCGGCCCAGAGCCGAGCCAAG
	Reverse	GCTGCCGCATGGGTTCTGACGG
p21p-up	Forward	AGTCTTGCCTGCCTTCAGAG
	Reverse	ACGAAGGGCTTGTTTTAGG
Human GAPDH promoter	Forward	AATGAATGGGCAGCCGTTAG
	Reverse	AGCTAGCCTCGCTCCACCTGAC

NOTE: Glyceraldehyde-3-phosphate dehydrogenase (GAPDH) was amplified as an internal control.
